# Conversations upon the Physical and Mental Hygiene of Girlhood

**Published:** 1881-01-20

**Authors:** T. S. Powell

**Affiliations:** Atlanta; Professor of Obstetrics and Diseases of Women, and Lecturer on Medical Ethics in the Southern Medical College


### Southern Medical Record.

EDITORS:

T. S. POWELL, M.D. W. T. GOLDSMITH, M.D. R. C. WORD, M.D.

*R. C. WORD, M.D., Managing Editor.*

☞All Communications and Letters on Business connected with the RECORD must be addressed to the Managing Editor.

VOL. XI. ATLANTA, GA., JANUARY 20, 1881. NO. 1.

### ORIGINAL AND SELECTED ARTICLES.

### CONVERSATIONS UPON THE PHYSICAL AND MENTAL HYGIENE OF GIRLHOOD.

BY T. S. POWELL, M.D.,

Professor of Obstetrics and Diseases of Women, and Lecturer on Medical Ethics in the Southern Medical College.

At the urgent solicitation of some of my partial friends, or those who, I fear, over-estimate the value of my experience in the treatment of diseases peculiar to the female pelvis, I have consented to publish a few cases from my case-book.

Some of these I may report in the usual manner, and others in the colloquial style, as in this way certain facts can be more clearly shown, and grave errors more effectually combatted.

To describe conditions and give remedial agents are not sufficient in every case. The why and how these symptoms and conditions arise must be fully understood, as they frequently determine the real cause to be very obscure.

To treat the diseases of females successfully, the physician should not be content with only a knowledge of their accumulative effects, but should thoroughly understand all their initial causative influences.

As most of the diseases of females have their origin in general anæmia, and in local hyperæmia, I will report first a few cases in that form, and in a dialogue, for the reasons to which I have referred.

Mrs. L. M., a very intelligent and cultivated lady, commenced this conversation by saying:

"Doctor, I have sent for you to talk with you about the condition of my daughter Mary. She has always been rather delicate, but we did not think her health sufficiently endangered to require special medical treatment, until about eighteen months ago. Since then she has been treated by several physicians; was attended by them consecutively before we came to this city; she has had no medicine, though, for five or six months, and I believe she is declining every day. We feel that something must be done to benefit her soon, or it will be too late, and we desire you to examine her condition carefully, Doctor, and tell me candidly if you can cure her."

Doctor—"Well, madam, I will give your daughter's case a careful investigation, but whether I can cure her or not, I do not know. You remember Moses lost the promised land by saying, 'I'—thus taking credit to himself that should be given to God.

"I have made it a rule, ever since I have been practicing medicine, never to say I could cure any one. I do, however, often refer to cases where patients have been restored to health, under my treatment, by learning the true cause of the disease, and using my best judgment in the adoption of remedies for its eradication. I am never satisfied with giving my patients only temporary relief. Dr. Rush said disease was a lawless enemy, and should be watched day and night. I think he is correct, especially in regard to female diseases. Physicians, therefore, should never experiment upon effects or symptoms, and allow causes to remain. To consider the symptoms of a disease alone is often a fatal error. These can never exist without a cause, and this cause I now propose to investigate in the case of your daughter, and, if possible, remove it at the earliest reasonable time. Suppose you bring Miss Mary in, and let us both talk with her frankly and cheerfully."

Mrs. M.—"Very well, Doctor; I will be glad to have you see her."

The young girl being called in and introduced, is received with the kindness and courtesy of a welcome and trusted friend.

Doctor—"And, now, Miss Mary, take a seat here near your mother and myself, so we can talk pleasantly to each other. Your mother and I have been speaking of you a good deal this morning."

Patient—"Yes, sir, I suppose so. Mamma has been very anxious about my health for some time, and not altogether without cause; yet I hope and believe I am not so ill as she seems to think I am."

Doctor—"That is true; and while your mother has great reason to feel anxious about your present condition and future health, I am glad to say that your general appearance to-day does not impress me with the opinion that your case is so very serious at present."

Mother—" Do I understand you to say, Doctor, that though Mary's condition is not so serious just now, it may become so in a short time, if not attended to properly ? "

Doctor—" Yes, madam, that was my meaning. How old are you, Miss Mary ? "

Patient—" I will be fifteen the 10th of next month."

Doctor—"Are you going to school at present ? "

Patient—" Yes, sir; I have not missed more than a month from school in several years, only during vacation."

Doctor—" Then you are fond of your studies ? "

Mother—" Yes, Doctor; until her health began to decline it was impossible to keep her from school, rain or sunshine. She was very ambitious, and generally stood first in her class. But for sometime she has been depressed in spirits at frequent intervals—seemed to become discouraged, and has fallen behind in her class."

Doctor—" How much do you walk every day, Miss Mary?"

Patient—" Not much—I have so little time. I walk to school in the morning and back in the afternoon. I then eat my dinner, study my French, or some other lessons, and then practice my music until about sunset."

Doctor—" I think I will soon discover the cause of all Miss Mary's ill-health, and the reason for these strange and unpleasant symptoms."

Mother—" I hope you will, Doctor ; and I think I begin to see myself where the difficulty lies. I am satisfied that my daughter's physical and mental capacity have both been overtaxed."

Doctor—" Yes, madam, that is very evident, and either will prevent a full and healthy development of body and mind. Many of our loveliest young girls, with brilliant prospects, die prematurely or live out miserable lives for the want of proper care and attention while passing from girlhood to the threshold of woman's maturity. From twelve to sixteen years is the most critical period in the life of every girl, and if she is not properly prepared for the very important transition, serious damage to both body and mind will be the result.

" It requires blood, muscle and nerve-force to effect perfectly the natural changes of that period, and these can only be secured by proper hygienic measures, such as nourishing food, enough bodily exercise of a suitable nature, plenty of refreshing sleep at night, and sufficient mental rest."

Mother—"If you are correct, Doctor, and I believe you are, it is not surprising that so many bright, happy girls die early, or live to become sickly women, and not perfectly developed in mind or body."

Doctor—"If I had the time, my dear madam, I could give you the history of a large number of cases that have come under my observation in the last thirty years, varied, of course, more or less, in symptoms and effects; but the distressing ill-health of all the patients, and the premature death of many, were caused by impoverishment of the blood, and consequent nervous exhaustion, both resulting from unnatural taxation of the physical and mental forces. The same unhappy results are also often brought about by unhealthy excitation of the emotional nature, which is produced by young girls being admitted too early into social circles, and allowed too much reading of sensational novels, love-sick stories, etc."

Mother—"If we mothers are too ignorant and thoughtless, Doctor, as I believe we are, to prevent these errors in the education and management of our daughters, do you not think that every family physician should so thoroughly understand his profession, and individually perform his duty, as to teach us how we can rear our children so as to avoid all these sad results, and instead to become perfectly developed in body, mind and spirit, as I believe the Creator intended they should be?"

Doctor—"I certainly do. But, my dear madam, in the first place, very few families, compared with former times, acknowledge that they have a family physician. They are too independent, and too much affected with the disease of the age for change and novelty. But in their frequent changes of a physician, if they do employ one who was instructed in the old school, where both head and heart were educated for the profession, and he feels it his duty to give such advice as will prevent illness, and most likely secure health, as well as prescribe the best remedies for the cure of disease, he is often laughed at, and set down as an old-fogy, unacquainted with the laws of modern society, and wanting in due deference to the opinions inculcated among so many of its members, and born of false pride and ignorance of some most vital questions. This state of things forces many of our skillful physicians to observe these laws of our social circles so detrimental to health, and to cultivate a desire to please where it is their first duty to instruct."

Mother—"I think you are again right, Doctor, and such a state of things in both society and schools is disgusting to sensible and intelligent persons. Our children had better have no education if it is to be gained at the loss of health, usefulness and happiness."

Doctor—"But these evils are not necessary accompaniments to the acquisition of even an accomplished education in its highest and truest meaning. The best and most thorough instruction can be acquired in perfect harmony with the laws of health, and fullest development of mind and body.

"When you ladies of fashion determine to conduct society upon Biblical principles and natural laws, you will find that its assemblies will be instructive and truly pleasurable, as well as conducive to the health and happiness of your daughters.

"I would make some explanatory remarks here in reference to this subject, but my engagements this morning will not permit me to do so. Let us return to Miss Mary's case."

Mother—"Very well, but I see she has left the room. Shall I call her again?"

Doctor—"No, madam, it is not necessary; I only wish to ask you a few more questions.

"How is your daughter's appetite?"

Mother—"Not good. It has not been for eight or ten months—ever since she ceased to menstruate. I have not been able to get anything she seems to relish, and of sufficient nourishment. Sometimes she has a great craving for things that we know are injurious to her health—such as pickles, preserves, vinegar, and similar things."

Doctor—"It is a self-evident fact, that if it requires blood and muscle to effect the transition from girlhood to womanhood, this important change cannot be successfully accomplished on preserves, pickles and candies, especially if the girl attempts to master mathematics, natural science, ancient and modern languages, music and other fashionable accomplishments."

Mother—"I have already occupied too much of your time, Doctor; but do tell me what is Mary's disease, and if you believe she can be cured?"

Doctor—"I do not think your daughter has any local organic disease, but her ill health is a general disorder of the physical functions, known by physicians as Anæmia, which means that the supply of nutrition is not sufficient to meet the wants and demands of the system, and bring about local development and the natural action of local functions. Thus you perceive that this general disorder is sometimes caused by the want of food suitable in quantity and quality, and sometimes by exhaustion of the nervous forces—the power that propels the blood in its natural channels throughout the entire system, conveying and distributing the nutrition necessary to maintain the vigor of each organ. Therefore, to be successful in removing the causes of this class, or expression of disease, the treatment must be made to give tone and strength to the digestive and assimilating organs."

"To do this a combination of such remedies must be used as is best calculated by its therapeutic effects, in connection with proper diet and exercise, to restore the general system to perfect order.

"As the first step towards the treatment, I will now give you this prescription:

"℞ Elixir of calizya........................................ ℥ ij

Pepsin................................................... ʒ ij

Tinct. nux vomica.......................................... ʒ i

Sub nit. bismuth........................................... ʒ i

Phosphate of iron......................................... gr. xvi

"M. Sig. Give one teaspoonful just before each meal in a wine glass of water. Also one or two tablespoonsful of saturated solution of chlorate of potash between meals.

"The solution is made by adding two ounces of potash to one pint of water."

Mother—"Is the medicine unpleasant to take?"

Doctor—"Not very. I do not think Miss Mary will complain of its taste, but I fear she will very much object to the rest of my prescription, and before I give it you must promise me to see that it is strictly carried out."

Mother—"I promise you I will, Doctor, for I am satisfied Mary cannot live long in her present state of health, and I am fully determined to have strict obedience to your prescription and instrutions."

Doctor—"I thank you, madam, and now give me your attention, if you please. First, your daughter must be taken from school and must also give up her music lessons until her health is restored; she must not be allowed to touch the piano. There is no exercise, either mental or physical, performed by young girls, that sooner or more certainly exhausts their nerve power than playing the piano too frequently and at too early an age. How often we see these little girls, even in their teens, with flat chest and undeveloped physical capacity, and hear them say: 'Oh! how my wrist and back ache! They feel like they will break to pieces.' It is torture, cruelty to confine a child or young girl at any task that causes mental or physical pain, and of course it is injurious to their health. It will ultimately destroy the bright and constant buoyancy of spirits, and the vigor and elasticity of body that should characterize children and young womanhood, and which is really their natural condition if they were born of healthy parents and have been properly bred from their early infancy."

Mother—"I never looked at it all in that light before, Doctor, and I begin to see more clearly how ignorant we parents are in regard to the proper rearing of our children, especially our daughters."

Doctor—"That is true, madam, but let us hope that parents will begin to educate themselves fully upon this subject.

"To return to my prescription. Your daughter must take more out door exercise; walk and ride more in the open air, not only for the muscular exercise, but that the whole system may receive a better and greater supply of oxygen. This will make more food necessary, and consequently richer blood will be made, and improved health will follow. Physical exercise can be of but little or no advantage if the mind be exhausted by study. It is an important truth that exercise *per se* does not increase muscular force but really exhausts its powers. Physical exercise is only beneficial to that extent which increases the appetite and aids digestion, so that the system will daily receive a large per cent. of strength, and the exhaustive process requisite to bring about this increase of appetite and activity of the digestive organs should be closely observed. That your daughter may be benefitted by outdoor exercise upon scientific principles, I would be glad if she could spend a few months in the country."

Mother—"Mary will like that, I am sure. She has an uncle living at a beautiful country home, and he has several daughters who are anxious for Mary to spend a month with them."

Doctor—"That is well, madam; let your daughter make the visit, if possible; and be careful to see that my advice, as to medicine, diet, exercise, etc., is strictly followed."

Mother—"You can rest assured, Doctor, that I will carry out your directions in every particular. If you are not in great haste, I would like to talk with you a little longer. I got the idea from some of your r remarks that many of the diseases or disorders of girls about the age of puberty, are caused by both body and mind being over-taxed before and during this critical period, and also by improper food and drinks, frequently in the way of unsuitable and hurtful condiments. Mary is not only very fond of pickles, sauces and vinegar, but uses more salt and pepper with her food than any child I ever saw. Do you think that is a natural taste, and is it hurtful or beneficial?"

Doctor—"Salt is a natural stimilant to the digestive organs, and a desire for a moderate quantity is not only a normal appetite, but is beneficial, and indeed indispensible. A small portion will promote digestion—an immoderate quantity will destroy it. I have seen reported a few distressing cases of indigestion that was supposed to result from the parties never using salt with their food, and on the other hand, I have known dreadful diseases produced by the use of salted meats. But it is proper to say in this connection, that perhaps it was not the salt itself in a healthful proportion that caused the disease, but the chemical change that had taken place in it, and produced by the combination of the salt and meat together.

"Why or how these chemical changes that make the meat unwholesome, frequently and positively poisons, is not known, but they are supposed to be caused sometimes by the meat being so often packed and unpacked, shipped and reshipped, and perhaps, also, to some extent, by the poisonous air of the storage rooms in which the meat remained so long, absorbing the impurities of an atmosphere that decaying vegetables and other matter made foul and noxious. In the country, the food is plain, sound, and in a natural state, consequently is more nutritious. The hams, and in fact, all meats, breads, vegetables, fruits, butter and milk are fresh and sweet—free from chemical changes—therefore, you seldom see meningitis, and cases like Miss Mary's among the country people."

Mother—"Doctor, you certainly don't mean to say that meningitis is caused by eating bad food?"

Doctor—"I do, madam. A large majority of the cases that have come under my observation I could trace to bad meal, spoiled meats, stale vegetables and poultry. In my native State, Virginia, the bread corn was selected and both ends were *nubbed*, a word I do not think is found in Webster's dictionary, but which means to shell off the unsound grains of corn on both ends of the cob for the pigs to eat, while the pure and fully matured grains were reserved for meal.

"No one was ever known to be sick from eating bread made of such meal as that. But in this fast age of progress the large ears of corn and the nubbins, the sound and the unsound, are all thrown in and shelled together, and ground into meal for the public. One-eighth of such meal is a slow but sure poison, often causing meningitis and other nervous diseases, that people have in *these days*.

"But what do the dealers care? People must have bread, and the dealers get their money all the same, poison or no poison."

Mother—"This is dreadful Doctor, but why do the proper authorities permit the sale of such articles of food?"

Doctor—"For the simple reason, madam, that for the last sixteen years, especially, our men have eaten so much tainted food they have neither the courage left to redress so great a wrong.

"They fear it will offend some of their *dear* constituents, who, perhaps, by virtue of their unlawful business, control forty or more floating votes.

"There will never be the proper legislation upon this question until the people inform themselves upon it intelligently in all its aspects, and then put men in authority who have proper views of the physical and moral welfare of the people, as well as their civil control and interest. No man can have these views and the principles to make laws for the health of the people, who wrangles for an office and desires to fill it only for its distinction and temporary emoluments. This, of course, madam, I say playfully, but still I think you find in it more truth than poetry.

"As is the custom in France, in every town and city in this country there should be a board of food inspectors, governed by stringent laws in regard to the food put upon the market for sale. Massachusetts has already set us the example in this important branch of legislation, and deserves honor from all the country for taking the initiatory movement. No human organs of digestion can make good nutrition out of unsound food. Do you know, madam, that it requires good food to make a good character as well as to give physical and mental power? This being the case, any intelligent person must see how very important it is that the food material sold to the public should be pure and wholesome. But I must leave you now, and these questions, until my next visit. I will call again in eight or ten days. Good morning, madam—I doubt not I shall find your daughter better and yourself in good spirits when we meet again."

(TO BE CONTINUED.)

